# Undervalued Pseudo-*nifH* Sequences in Public Databases Distort Metagenomic Insights into Biological Nitrogen Fixers

**DOI:** 10.1128/msphere.00785-21

**Published:** 2021-11-17

**Authors:** Kazumori Mise, Yoko Masuda, Keishi Senoo, Hideomi Itoh

**Affiliations:** a National Institute of Advanced Industrial Science and Technology (AIST) Hokkaido, Sapporo, Hokkaido, Japan; b Department of Applied Biological Chemistry, Graduate School of Agricultural and Life Sciences, The University of Tokyo, Tokyo, Japan; c Collaborative Research Institute for Innovative Microbiology, The University of Tokyo, Tokyo, Japan; U.S. Department of Energy Joint Genome Institute

**Keywords:** bioinformatics, computational biology, diazotrophs, genomics, metagenomics, nitrogen fixation

## Abstract

Nitrogen fixation, a distinct process incorporating the inactive atmospheric nitrogen into the active biological processes, has been a major topic in biological and geochemical studies. Currently, insights into diversity and distribution of nitrogen-fixing microbes are dependent upon homology-based analyses of nitrogenase genes, especially the *nifH* gene, which are broadly conserved in nitrogen-fixing microbes. Here, we report the pitfall of using *nifH* as a marker of microbial nitrogen fixation. We exhaustively analyzed genomes in RefSeq (231,908 genomes) and KEGG (6,509 genomes) and cooccurrence and gene order patterns of nitrogenase genes (including *nifH*) therein. Up to 20% of *nifH*-harboring genomes lacked *nifD* and *nifK*, which encode essential subunits of nitrogenase, within 10 coding sequences upstream or downstream of *nifH* or on the same genome. According to a phenotypic database of prokaryotes, no species and strains harboring only *nifH* possess nitrogen-fixing activities, which shows that these *nifH* genes are “pseudo”-*nifH* genes. Pseudo-*nifH* sequences mainly belong to anaerobic microbes, including members of the class *Clostridia* and methanogens. We also detected many pseudo-*nifH* reads from metagenomic sequences of anaerobic environments such as animal guts, wastewater, paddy soils, and sediments. In some samples, pseudo-*nifH* overwhelmed the number of “true” *nifH* reads by 50% or 10 times. Because of the high sequence similarity between pseudo- and true-*nifH*, pronounced amounts of *nifH*-like reads were not confidently classified. Overall, our results encourage reconsideration of the conventional use of *nifH* for detecting nitrogen-fixing microbes, while suggesting that *nifD* or *nifK* would be a more reliable marker.

**IMPORTANCE** Nitrogen-fixing microbes affect biogeochemical cycling, agricultural productivity, and microbial ecosystems, and their distributions have been investigated intensively using genomic and metagenomic sequencing. Currently, insights into nitrogen fixers in the environment have been acquired by homology searches against nitrogenase genes, particularly the *nifH* gene, in public databases. Here, we report that public databases include a significant amount of incorrectly annotated *nifH* sequences (pseudo-*nifH*). We exhaustively investigated the genomic structures of *nifH*-harboring genomes and found hundreds of pseudo-*nifH* sequences in RefSeq and KEGG. Over half of these pseudo-*nifH* sequences belonged to members of the class *Clostridia*, which is supposed to be a prominent nitrogen-fixing clade. We also found that the abundance of nitrogen fixers in metagenomes could be overestimated by 1.5 to >10 times due to pseudo-*nifH* recorded in public databases. Our results encourage reconsideration of the prevalent use of *nifH* as a marker of nitrogen-fixing microbes.

## INTRODUCTION

Microbial nitrogen fixation is a prominent process in biogeochemical cycling, and the ecology and evolution of nitrogen-fixing microbes have received extraordinary attention from researchers in various academic fields. While certain clades of bacteria, including cyanobacteria, *Clostridium*, azotobacter, and legume symbionts, are known for their diazotrophic activities (1), recent genomic and metagenomic surveys have unveiled unexpected diversity among the distributions of diazotrophic communities on Earth (2, 3). Insights into the drivers of nitrogen fixation in the environment are of interest in microbial physiology, ecology, and agriculture, and they are useful in modeling and predicting the dynamics of nitrogen cycling (4, 5). Importantly, nitrogen fixation in gut symbionts has been linked to nitrogen acquisition by the host, which has led to much attention in animal biology studies (6).

A key approach to successful (meta)genomic studies is the use of conserved “core” genes that are essential for nitrogen fixation. Nitrogen fixation is exclusively driven by nitrogenases, and diazotrophic microbes commonly harbor a distinct set of genes, including typical (e.g., *nifH*, *nifD*, and *nifK*) and atypical (e.g., *vnfD* and *anfD*) genes, that encode nitrogenase subunits (7). These nitrogenase genes have been regarded as the hallmarks of diazotrophs in genomic and metagenomic analyses.

Particularly popular among these markers is *nifH*. *nifH* is a gene encoding an Fe protein named nitrogenase reductase (NifH), which constitutes a subunit of nitrogenase (8). It should be noted that NifH does not directly interact with N_2_ molecules; rather, it reduces other subunits constituting nitrogenase, namely, NifD/NifK subunits, that catalyze the cleavage of the N–N triple bond. The prevalent use of *nifH* is presumably attributed to the development of the first degenerative primers for PCR amplification of *nifH* (9). The use of these primers for fingerprinting (e.g., PCR denaturing gradient gel electrophoresis), quantitative PCR, and amplicon sequencing analyses has substantially expanded scientific knowledge about the diversity of diazotrophic prokaryotes (10–12).

While the straightforward relationship between function and gene presence/absence is useful, it may be not be the case for *nifH*. Previous studies have suggested that some *nifH* genes are not involved in nitrogen fixation (13). For example, a group of *nifH* homologs, named cluster IV (or group IV), belong to nondiazotrophic methanogens, whereas another group of *nifH* homologs, called cluster V (or group V), include protochlorophyllide reductase or chlorophyllide reductase genes (14). In addition, only *nifH* homologs have been detected in the genomes of some methanogenic archaea, while *nifD* and *nifK* are not (15). These data challenge the long-established conception that *nifH* is a primary hallmark of diazotrophic potential. In addition, it is speculated that use of *nifH* as a biomarker would lead to an overestimation of the abundance and diversity of diazotrophic microbes, as well as biased estimation of diazotrophic community structures. Nevertheless, quantitative analysis for such “pseudo-*nifH*” has been scarcely done, and therefore little is known about how prevalent pseudo-*nifH* sequences are in public genomic databases and how this affects metagenomic insights into diazotrophic microbiomes.

To quantify distribution of these “pseudo-*nifH*” genes among prokaryotic genomes and metagenomes, the boundary between “true-*nifH*” (i.e., contributing to nitrogen fixation) and pseudo-*nifH* needs to be better clarified. A genome-oriented analysis might provide a way to determine the distribution. For example, a *nifH* sequence without other genes constituting nitrogenase (e.g., *nifD*, *nifK*) in its neighborhood might be a pseudo-*nifH*. Moreover, if no other nitrogenase gene exists on the genome, that *nifH* is likely a pseudo-*nifH* (note that NifH does not directly cleave the N–N triple bond; therefore, NifH alone cannot modulate nitrogen fixation). These kinds of predictions that are based on neighboring genes and coexisting genes on the genomes have been versatile approaches in gene functional annotations (16–18) that complement the conventional homology search.

In this work, we questioned the suitability of *nifH* as a hallmark of diazotrophs. We aimed to elucidate the distribution of true- and pseudo-*nifH* among prokaryotic genomes and environmental metagenomes. First, we applied gene coexistence/neighborhood analyses to *nifH*-harboring genomes stored in highly reputed public databases (i.e., RefSeq and Kyoto Encyclopedia of Genes and Genomes [KEGG]). After confirming the accuracy of our method by checking the consistency with previous isolation-based reports, we further examined the distribution of true- and pseudo-*nifH* in environmental metagenomes. Finally, we discussed the possible outcomes from prevalent pseudo-*nifH* stored in public databases and metagenomes.

## RESULTS AND DISCUSSION

### Cryptic distribution of *nifH* in publicly available prokaryotic genomes.

To search for the candidates of pseudo-*nifH*, we first analyzed the distribution of nitrogenase genes in two fundamental, well-annotated, and high-quality genome databases, namely, National Center for Biotechnology Information (NCBI) RefSeq (19) and KEGG (20). Here, we were able to observe a cryptic distribution of nitrogen fixation genes.

**FIG 1:**
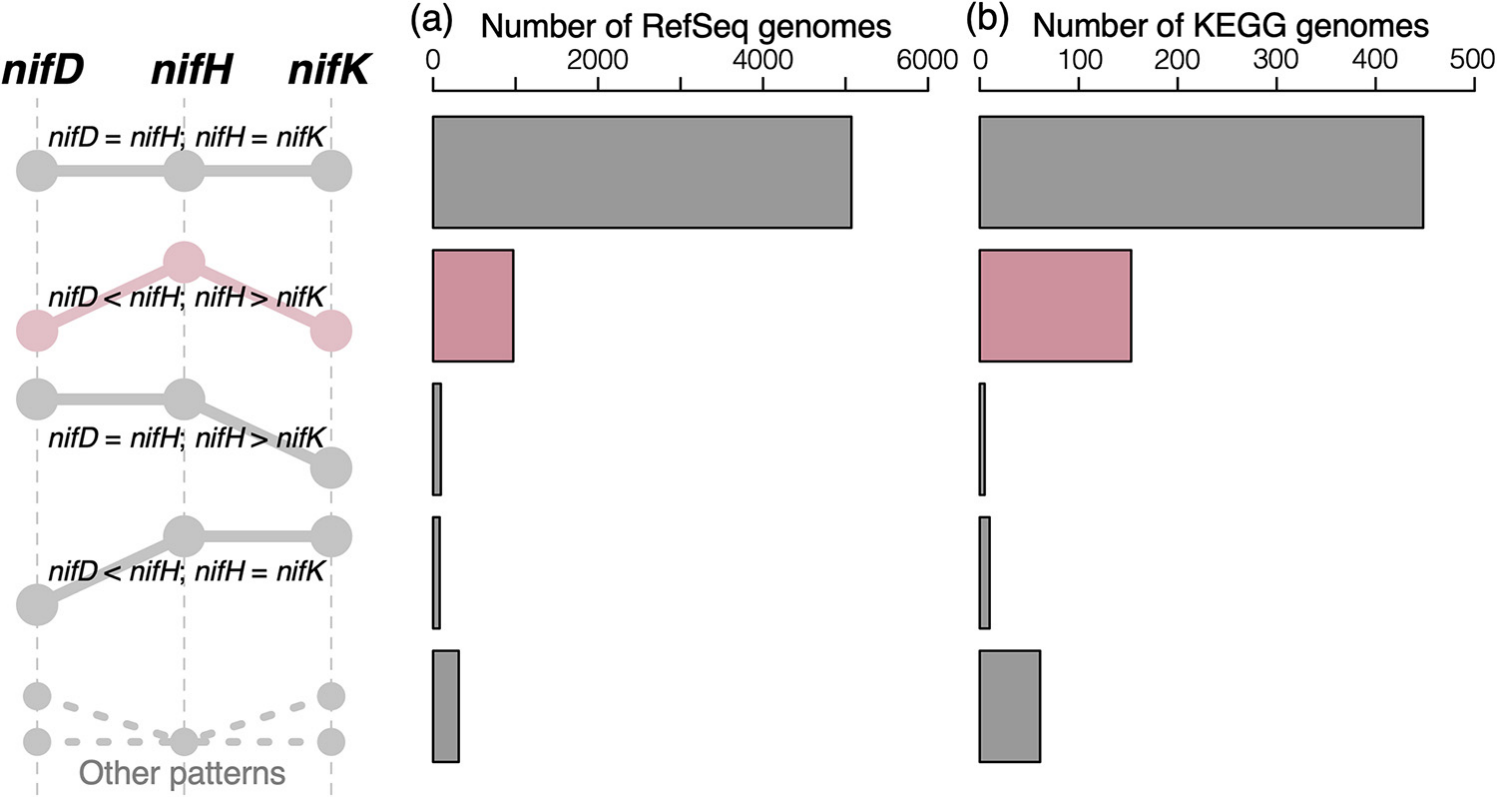
Numbers of RefSeq (a) and KEGG (b) genomes harboring an equal/unequal number of nitrogenase genes. (Left) The line plot shows (im)balances between the copy numbers of *nifH*, *nifD* (including *vnfD* and *anfD*), and *nifK* (including *vnfK* and *anfK*). The first row indicates genomes harboring an equal copy number of *nifH*, *nifD*, and *nifK*. The four lower rows represent genomes with an unequal copy number of the three genes. Note that genomes with excessive *nifH* are remarkably abundant, as indicated by the second row (pink bars and line plot).

Among the 231,908 RefSeq genomes analyzed, 6,529 genomes (excluding ones with the completeness below 95%) harbored one or more coding sequences (CDSs) annotated either as *nifH*, *nifD*, *nifK*, *vnfD*, *vnfK*, *anfD*, or *anfK*. Here, we accounted for atypical nitrogenase genes, *vnf* and *anf* (21), because *nifH* is homologous to *vnfH* and *anfH* and they may be confused in RefSeq (of note, no CDS was annotated as *vnfH* or *anfH*). In fact, we observed 66 genomes with *nifH* neighboring with *vnfD* or *vnfK* and 12 genomes with *nifH* neighboring with *anfD* or *anfK* (examples are shown in Table S1 in the supplemental material). While many of the genomes had the same copy numbers of *nifH*, *nifD* (including *vnfD* and *anfD*), and *nifK* (including *vnfK* and *anfK*), 1,457 genomes (22.3% of the 6,529 genomes) harbored an unequal number of these genes (Fig. 1a). The copy number of *nifH* was higher than those of *nifD* and *nifK* in 972 genomes (66.7% of the 1,457 genomes), and 373 genomes (25.6% of the 1,457 genomes) had only *nifH*. In contrast, genomes lacking *nifH* but possessing *nifD* or *nifK* were quite rare (96 genomes, 6.59% of the 1,457 genomes). These imbalanced results clearly conflict with the well-established conception that *nifH*, *nifD*, and *nifK* together constitute a gene cluster (*nif* operon) serving for nitrogen fixation (14). Therefore, the link between *nifH* and nitrogen fixation might not exist.

10.1128/msphere.00785-21.1 TABLE S1 Examples of syntenies consisting of *nifH* and *vnf* or *anf* genes in RefSeq. Download Table S1, DOCX file, 0.1 MB. Copyright © 2021 Mise et al. 2021 Mise et al. https://creativecommons.org/licenses/by/4.0/ This content is distributed under the terms of the Creative Commons Attribution 4.0 International license.

Using the hidden Markov model (HMM)-based method targeting *nifD/vnfD/anfD* and *nifK/vnfK/anfK* (22), we reannotated the CDSs of the 6,529 genomes (see above) to rule out the possibility that *nifD* and *nifK* had been overlooked by NCBI’s in-house annotation protocol (PGAP) (19). Note that the annotations in RefSeq bear some inconsistency (variation) even within closely related homologs (for example if the concept of Gene Ontology [23, 24] is recalled), which called for this kind of reannotation. The HMM is suitable for minimizing false-negative results as it is typically more sensitive than BLAST-like algorithms or software (including PGAP) (25). More CDSs were annotated as *nifD* or *nifK* by HMM, including those annotated otherwise in RefSeq: of the 373 genomes harboring only *nifH* according to RefSeq annotations, 136 (36.4% of the 373 genomes) turned out to possess at least one of the *nifD* (including *vnfD* and *anfD*) or *nifK* (including *vnfK* and *anfK*) gene. Nevertheless, *nifH* remained more prevalent than the other genes in question. The lack of *nifD*/*nifK* could be partially attributed to the incompleteness or assembly errors of the genomes; however, their effects would be mostly negligible considering the rigorous quality control procedure of these databases and our in-house filtering of low-completeness (i.e., <95%) genomes.

The KEGG database, where orthologous groups are manually defined based upon a rigorous literature survey, also presented excessive prevalence of *nifH* (Fig. 1b) in addition to the RefSeq database. Among the 6,509 prokaryotic genomes analyzed, 677 contained one or more of nitrogenase orthologs (excluding ones with the completeness below 95%), namely, *nifD*/*anfD* (K02586), *nifH* (K02588), *nifK*/*anfK* (K02591), *vnfD* (K22896), and *vnfK* (K22897). *nifH* (K02588) was distributed in 669 genomes, 72 (10.8%) of which were not concomitant with any of the other nitrogenase orthologs. On the other hand, genomes harboring *nifD/vnfD/anfD* or *nifK/vnfK/anfK* but lacking *nifH* (K02588) were rare (four genomes, 0.6%).

**FIG 2:**
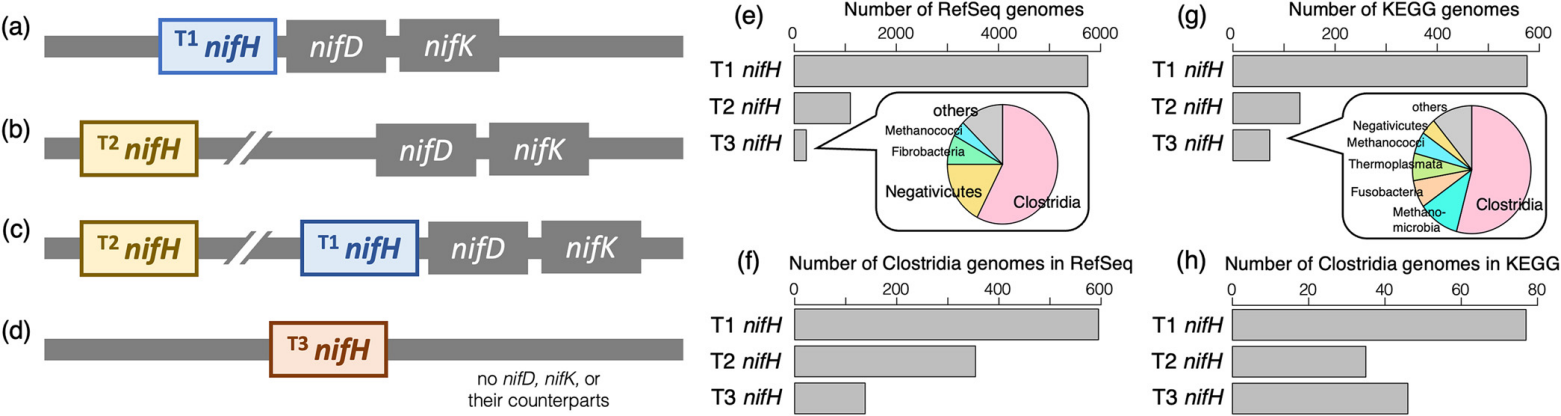
Illustration of three types of *nifH* and their distributions in RefSeq and KEGG. (a) *nifH* accompanied by *nifD* (including *vnfD* and *anfD*) or *nifK* (including *vnfK* and *anfK*) in its neighborhood is called T1-*nifH*. (b) *nifH* accompanied by *nifD* or *nifK*, not in the neighborhood but somewhere distant on the same genome, is called T2-*nifH*. (c) T1- and T2-*nifH* might coexist on one genome. (d) *nifH* on a genome lacking *nifD* and *nifK* is called T3-*nifH*. (e) Number of RefSeq genomes harboring T1-, T2-, and T3-*nifH*. The pie chart shows the taxonomic composition of genomes with T3-*nifH*. (f) Number of RefSeq genomes belonging to the class *Clostridia* that harbor T1-, T2-, and T3-*nifH*. (g) Number of KEGG genomes harboring T1-, T2-, and T3-*nifH*. The pie chart shows the taxonomic composition of genomes with T3-*nifH*. (h) Number of KEGG genomes belonging to the class *Clostridia* that harbor T1-, T2-, and T3-*nifH*.

Importantly, we observed three types of *nifH* CDS on RefSeq/KEGG genomes, which are hereafter called T1-, T2-, and T3-*nifH* (Fig. 2a to d). T1-*nifH* is accompanied by at least one of the orthologs encoding nitrogenase subunits (namely, *nifD*, *nifK*, *vnfD*, *vnfK*, *anfD*, and *anfK*) in their neighborhood (not more than 10 CDSs away from *nifH*). T1-*nifH* likely constitutes a nitrogen fixation operon that plays a role in nitrogen fixation. T2-*nifH* is not accompanied by the above-mentioned nitrogenase subunits in their neighborhood, but one or more exist elsewhere on the genome (including plasmids). This type of *nifH* is somewhat elusive: it appears to be different from the typical structure of the nitrogen fixation operon (14, 26), but it might work in cooperation with other subunits that are encoded distantly (27). T3-*nifH* is a “stand-alone” type of *nifH*, meaning no other nitrogenase genes exist in the genome or plasmids. It should not function as nitrogenase reductase (NifH) because of lack of the relevant nitrogenase (NifDK), if no orthologs were overlooked (either by genomic incompleteness or annotation failure). NifH is a member of the ATPase superfamily (28): NifH binds to and hydrolyzes ATP along with transferring electrons to nitrogenase (29). Therefore, T3-NifH may function as some kinds of ATPase by itself. Note that some genomes harbor both T1-*nifH* and T2-*nifH*, whereas T3-*nifH* and the other two are mutually exclusive by definition. The number of RefSeq and KEGG genomes having each type of *nifH* are summarized in Fig. 2e and g, respectively.

### Genome-based distinction between T1/T2- and T3-*nifH* is consistent with experimentally validated diazotrophic capability of each species.

Next, we intended to investigate whether our three-class classification of *nifH* is in line with collective insights into species-level diazotrophic activities reported in numerous previous reports. For this purpose, we referred to FAPROTAX (30), which is a manually curated database that bridges prokaryotic taxonomy names with their functions (including nitrogen fixation). Items in FAPROTAX have been manually propagated from acknowledged and reliable literature sources, such as *Bergey’s Manual of Systematic Bacteriology* (*Bergey’s Manual*) and the *International Journal of Systematic and Evolutionary Microbiology*. A key feature of FAPROTAX is that it is not dependent on genomic sequences. The insights into diazotrophy have not necessarily been coupled with whole-genome sequencing, and therefore, strict correspondence between diazotrophic activity and whole-genome sequences is not available. This warrants the prediction of diazotrophic activity via taxonomic names. Note that FAPROTAX is conceptually much different from PICRUSt, which estimates functional gene profiles from the available genomes of extant prokaryotes, using 16S rRNA gene sequences as the key (31). The phenotype-oriented (rather than genome-oriented) feature of FAPROTAX enabled us to speculate the diazotrophic activities of T1 *nifH*- and T3 *nifH*-harboring prokaryotes.

FAPROTAX included approximately 200 records of nitrogen-fixing prokaryotes. Of the 5,749 and 576 prokaryotic strains harboring T1-*nifH* in RefSeq, 1,600 (27.8%) matched 200 records in FAPROTAX (Table 1). Of the 448 strains harboring T2-*nifH* but no T1-*nifH*, 337 (75.2%) were assigned as nitrogen-fixing microbes. Such a high proportion of hits among T2-*nifH* should be attributed to the taxonomic composition of T2-*nifH*-harboring genomes. They consisted of long-known and well-characterized diazotrophs, especially *Bradyrhizobium*, *Rhizobium* (251 and 39 of 448 strains, respectively). In fact, a previous study provides direct evidence for the diazotrophic activity of T2-*nifH*-harboring *Bradyrhizobium* (27). On the other hand, none of the 236 strains harboring T3-*nifH* overlapped with 200 records of FAPROTAX (Table 1). Genomes in KEGG also showed overall similar trends, although one of the T3-*nifH*-harboring strains (Methanospirillum hungatei strain JF-1) was exceptionally estimated to be capable of nitrogen fixation (Table 1). This conflict can be explained by within-species diversity of M. hungatei: another strain, GP1, has been shown to fix nitrogen (32), and FAPROTAX has been built upon this knowledge (33). Of note, the genome of strain GP1 (GCF_019263745.1 in RefSeq) bears a T1-*nifH* accompanied by *nifD.* On the other hand, the diazotrophic activity of strain JF-1 has not been reported to the best of our knowledge.

**TABLE 1:** Numbers of prokaryotic species and strains harboring T1-*nifH*, T2-*nifH* but no T1-*nifH*, and T3-*nifH* on the genomes from RefSeq and KEGG, with or without a previous report on diazotrophic activity

Database	Strain or species chararacteristic	No. of prokaryotic species and strains	
		Diazotrophic activity reported	No diazotrophic activity reported/not yet investigated
RefSeq	Harboring T1-*nifH* on their genomes	1,600	4,149
	Harboring T2-*nifH* but not T1-*nifH* on their genomes	337	111
	Harboring T3-*nifH* on their genomes	0	236
KEGG	Harboring T1-*nifH* on their genomes	132	444
	Harboring T2-*nifH* but not T1-*nifH* on their genomes	14	6
	Harboring T3-*nifH* on their genomes	1	71

It should be noted that FAPROTAX is not an exhaustive database covering all lineages of prokaryotes. That is, it should be commonplace that strains unlisted in FAPROTAX are capable of nitrogen fixation. In addition, as the aforementioned exception suggests, microdiversity in nitrogen-fixing capabilities, which is beyond the resolution of FAPROTAX, could lead to partially inaccurate estimation. Nevertheless, such microdiversity would not override the stark contrast between T1/T2 (T1/2)- and T3-*nifH*, which is observed in multiple distinct linages. Overall, the present result is unlikely to contradict our expectation that genome-based distinction between T1- and T3-*nifH* reflects the presence/absence of strain-level nitrogen fixation capability. In addition, T2-*nifH* genes are likely to be involved in nitrogen fixation.

Previously, several studies have described the existence of pseudo-*nifH* or T3-*nifH*, in line with our results. In particular, some methanogens such as *Methanobrevibacter*, *Methanocaldococcus*, and *Methanosarcina* have been reported to harbor T3-*nifH* (15) or uncharacterized *nifH* homologs (13, 14). Part of these genes have been later characterized as coenzyme F430 biosynthesis genes (34). Another example of confusing *nifH* homologs are protochlorophyllide reductase genes among *Cyanobacteria* that are serving for biosynthesis of chlorophyll (35). While these gene products are functionally similar to NifH, they have been annotated as such in RefSeq and KEGG, and therefore, they were not included in our analysis.

### True- and pseudo-*nifH* genes are not discernible by short-read sequences or predicted molecular structures.

Among the 236 RefSeq genomes harboring T3-*nifH* (i.e., genomes without *nifDK*/*vnfDK*/*anfDK*), 136 (57.6%) belonged to *Clostridia* (Fig. 2e). Notably, *Clostridia* include long-known diazotrophic bacteria, such as *Clostridium* spp. In fact, 586 and 351 RefSeq genomes belonging to *Clostridia* possessed T1-*nifH* and T2-*nifH*, respectively (Fig. 2f). Other prokaryotic clades, such as the class *Negativicutes* and methanogens, also possessed all three types of *nifH*. The KEGG genomes presented a similar distribution of T3-*nifH* (Fig. 2g and h).

**FIG 3:**
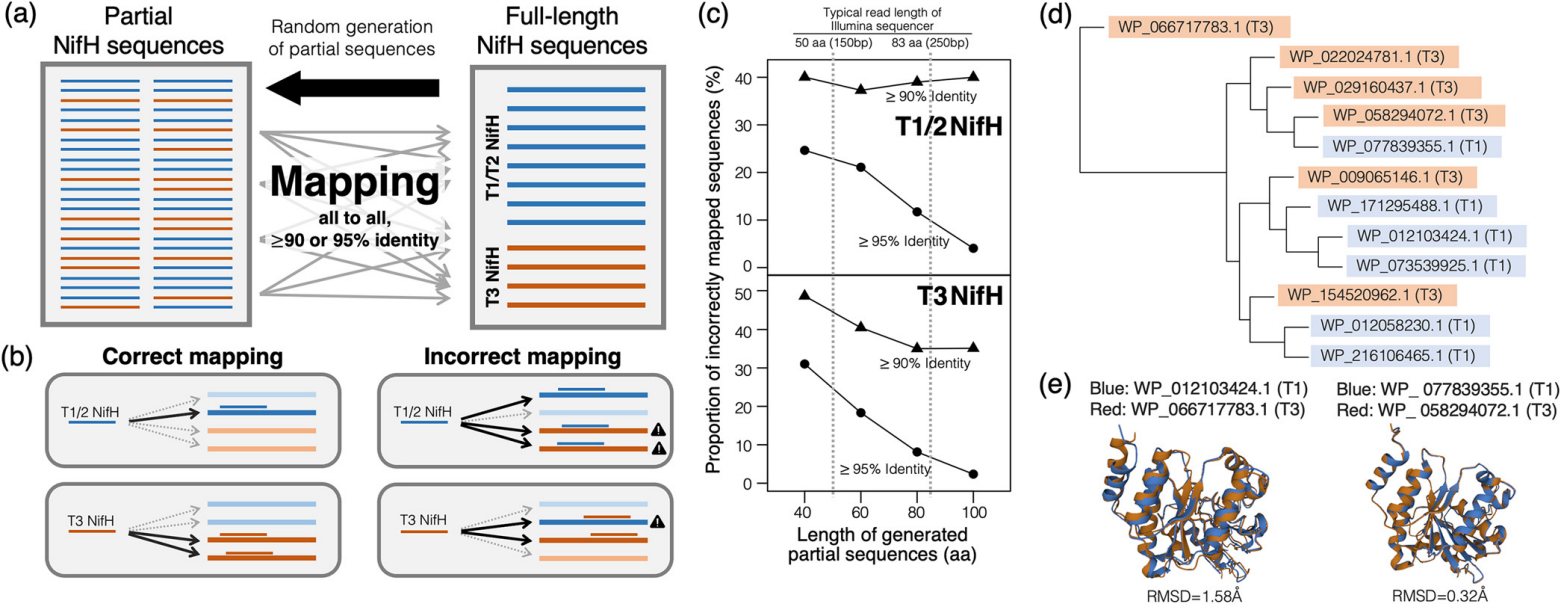
Proximity between T1/2-NifH and T3-NifH. (a) Schematic diagram showing the generation of partial NifH sequences and their mapping on full-length NifH sequences. (b) Classification between correct mapping and incorrect mapping of partial NifH sequences. If partial T1/2-NifH was mapped only on full-length T1/2-NifH, the mapping was regarded as correct. If it was mapped on T3-NifH (in addition to T1/2-NifH), the mapping was regarded as incorrect. (c) Proportion of incorrect mapping with different query lengths and identity thresholds. Gray dotted lines indicate 50 aa and 83 aa, corresponding to 150 bp and 250 bp on DNA, respectively, which are typical read lengths of Illumina short-read sequencers. (d) Cluster dendrogram showing similarities between the protein structures of T1- and T3-NifH. A RefSeq accession number, as well as a type of NifH (T1 or T3), is indicated for each node. Nodes for T1- and T3-NifH are highlighted in blue and red, respectively. (e) Two examples of protein structural alignments between T1- and T3-NifH. RefSeq accession numbers of subjected NifH sequences, as well as the RMSD between two NifH, are indicated. The visualizations were generated on the PDB’s web server.

Given this, we questioned whether the biological sequences of T1/2- and T3-*nifH* can be differentiated (especially based on partial sequences generated by high-throughput sequencers). We randomly generated partial sequences of NifH (40, 60, 80, and 100 amino acids [aa], corresponding to 120 to 300 bases, which cover the range of typical read lengths from Illumina sequencers) (Fig. 3a) and mapped them onto the full-length NifH at a similarity threshold of 90% or 95% (Fig. 3b). A number of query sequences were mapped “incorrectly,” i.e., partial sequences of T1-NifH were mapped to T3-NifH or vice versa (35.0 to 48.6% and 2.4 to 31.0% when the sequence similarity threshold was set at 90% and 95%, respectively). As expected, the proportion of incorrect mapping became larger when the query sequences were shorter or the similarity threshold for mapping was lower (Fig. 3c). This suggests that T1-NifH and T3-NifH are often not distinguishable from their partial sequences that can be generated by high-throughput sequencers. That said, removing T3-NifH sequences from the reference database might not improve the specificity of *nifH* detection in short-read shotgun metagenomic analyses.

We also compared molecular structures of T1-NifH and T3-NifH by using AlphaFold2, a state-of-the-art molecular structure predictor (36). We quantified structural differences between T1-NifH and T3-NifH using root mean square deviations (RMSDs). Structural differences between T1-NifH and T3-NifH were minor compared with those within T1-NifH or within T3-NifH (Fig. 3d). RMSDs were overall less than 2 Å, and pairwise structural alignments presented highly conserved secondary structures (Fig. 3e). These features indicated the close functional and evolutionary relationship between true and pseudo-NifH sequences.

Furthermore, we investigated sequence domains and regions that are highly conserved or divergent between T1/2- and T3-NifH. We constructed a multiple sequence alignment (MSA) of T1/2- and T3-NifH sequences (356 aa) and picked 40 column-long subsequences from the MSA (see Fig. S1b in the supplemental material; each subsequence may include several gaps). Then we used sequence similarity networks to evaluate the distinguishability between the subreads of T1/2- and T3-NifH, where each sequence was classified as either a “distinct” or “confusing” sequence (Fig. S1a). The proportion of confusing sequences were quite different in different regions. More specifically, the subsequences from the N end and middle regions of the MSA (30 to 70 and 151 to 230 aa) were confusing, while the C-end subsequences were mostly distinct. This indicates that the N end and middle regions are highly conserved between T1/2- and T3-NifH. In agreement with this, the middle regions (151 to 230 aa) include the ligand-binding site of the NifH molecules (37). Furthermore, many pairs of *nifH* universal primers have been designed targeting the upper region of *nifH* genes (38), which are rather conserved between T1/2- and T3-*nifH*.

10.1128/msphere.00785-21.4 FIG S1 Similarity network analysis of partial sequences of T1/2- and T3-NifH. (a) The conceptual diagram of network analysis employed here. Each node represents one NifH sequence, and any pair of similar sequences is connected by an edge. T1/2-NifH (blue nodes) that are not directly connected to T3-NifH are “distinct T1/2-NifH” (as they are clearly distinct from T3-NifH), whereas T3-NifH (orange nodes) without direct connection to T1-NifH are “distinct T3-NifH.” When a pair of T1/2- and T3-NifH sequences are highly similar and directly connected, these sequences are easily confused with the other type of NifH and therefore regarded as “confusing T1/2-NifH” or “confusing T3-NifH.” (b) The proportions of distinct/confusing T1/2- and T3-NifH sequences. For each of the 40-base regions taken from the multiple sequence alignment (MSA) of NifH, partial T1/2- and T3-NifH sequences were fed into network analysis (described above for panel a) and classified into “distinct” or “confusing” sequences. Note that sparse regions of MSA (regions mostly occupied with gaps) were not used. The two rows of bar plots differ in the sequence similarity threshold used for network analysis: 95% for the middle row and 90% for the bottom row. Download FIG S1, TIF file, 2.1 MB. Copyright © 2021 Mise et al. 2021 Mise et al. https://creativecommons.org/licenses/by/4.0/ This content is distributed under the terms of the Creative Commons Attribution 4.0 International license.

### Impact of prevalent pseudo-*nifH* on metagenomic analyses.

To elucidate the impact of pseudo-*nifH* sequences on shotgun metagenomic analyses, we reanalyzed the publicly available short-read metagenomic sequences. Because many of the genomes harboring T3-*nifH* are affiliated with anaerobes, we predicted that metagenomic analyses of anaerobic environments were subject to pseudo-*nifH* errors in the reference database. We obtained and processed shotgun metagenomic data sets from sludge, wastewater, human gut, termite gut, paddy soil, and sediment (Table S2) and then counted the number of *nifH*, *nifD*, and *nifK* sequences contained therein.

10.1128/msphere.00785-21.2 TABLE S2 Metagenomic data sets used in this study. Download Table S2, DOCX file, 0.09 MB. Copyright © 2021 Mise et al. 2021 Mise et al. https://creativecommons.org/licenses/by/4.0/ This content is distributed under the terms of the Creative Commons Attribution 4.0 International license.

**FIG 4:**
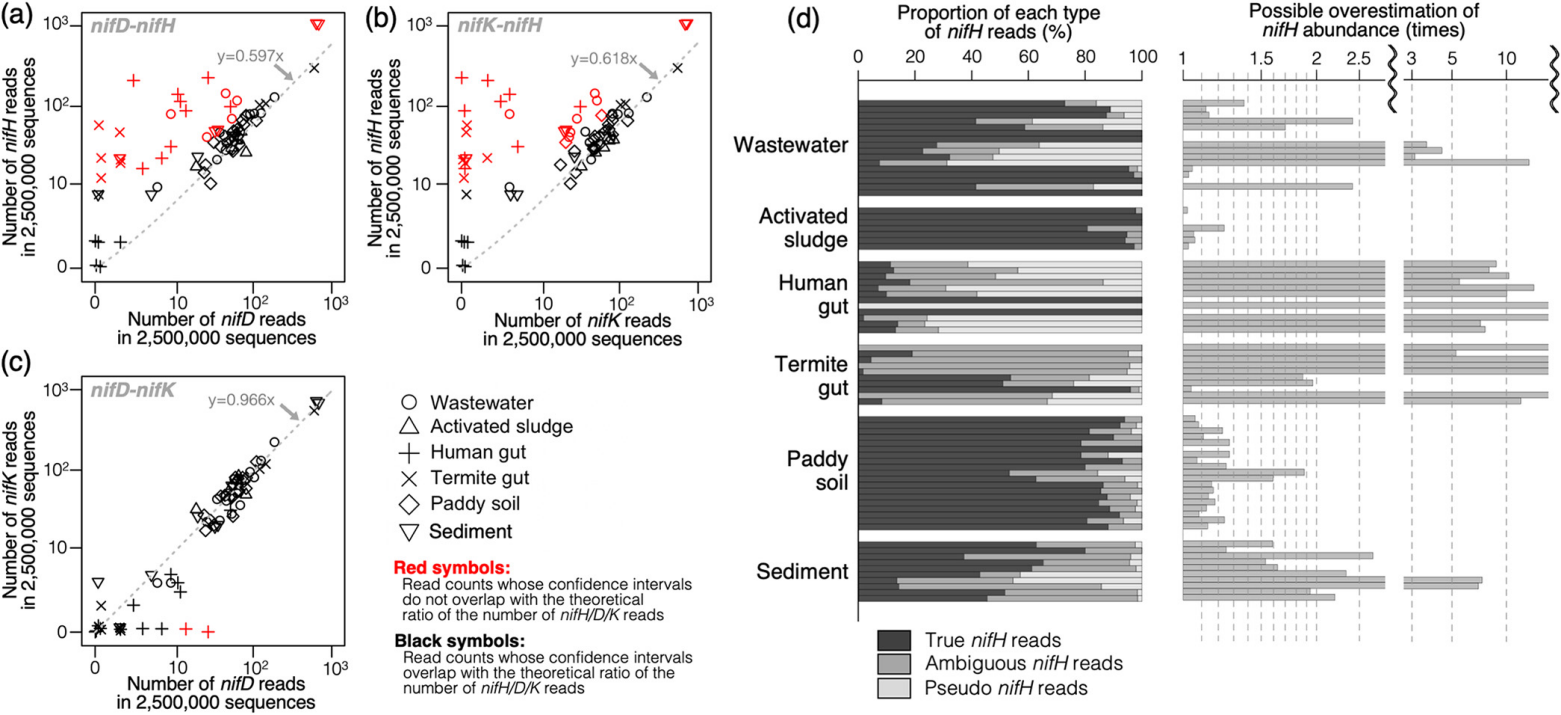
The outcome of focusing on *nifH* in shotgun metagenomic studies. (a) The relationship between read counts of *nifD* and *nifH*. The *x* and *y* axes are displayed in logarithmic scale [log (1 + *x*)]. The position of each point is slightly jittered to mitigate overlap between points [especially around (0,0)]. The gray dotted line indicates the theoretical relationship between read counts of two genes, where the number of *nifD* reads and *nifH* reads are proportional to the whole gene lengths of *nifD* and *nifH* (894 and 1,497 bp, respectively; the ratio is 0.597). Points statistically deviating from the theoretical proportion (gray dotted line) are colored red (see also Fig. S2 in the supplemental material). (b) Relationship between read counts of *nifK* and *nifH*. (c) Relationship between read counts of *nifD* and *nifK*. (d) The left panel shows proportions of true, ambiguous, and pseudo-*nifH* reads. The right panel shows the reciprocal of the proportion of true-*nifH* reads. This value represents the degree in which *nifH* abundance is possibly overestimated owing to pseudo-*nifH* reads. The horizontal axis is displayed in logarithmic scale.

As expected, we found that the number of *nifH* reads were excessive compared with those of *nifD* and *nifK* (Fig. 4a and b). Of note, the lengths of *nifH* sequences are typically shorter than those of *nifD* and *nifK* (Table S3), so the differences in read counts cannot be attributed to differences in gene length (Fig. 4a and b; Fig. S2a and b). On the other hand, the number of reads annotated as *nifD* and *nifK* were proportional (Fig. 4c; Fig. S2c). Only two outlier samples, where *nifD* reads were abundant but *nifK* reads were absent (Fig. 4c), contained reads similar to *nifD* of Phascolarctobacterium faecium or *Selenomonas* spp., which possessed only *nifD* and no *nifK*. Overall, considering the extensive prevalence of pseudo-*nifH* among prokaryotic genomes, our results indicated that *nifD* and *nifK* are relatively reliable markers of nitrogen-fixing microbes, whereas *nifH* is not.

10.1128/msphere.00785-21.3 TABLE S3 Lengths of three nitrogenase genes (and their products), namely, *nifH* (NifH), *nifD* (NifD), and *nifK* (NifK), in KEGG. Average and quartile lengths (quartile 1 [Q1] to Q3) are indicated. Nucleotide sequence lengths were calculated by tripling the amino acid sequence length. Download Table S3, DOCX file, 0.1 MB. Copyright © 2021 Mise et al. 2021 Mise et al. https://creativecommons.org/licenses/by/4.0/ This content is distributed under the terms of the Creative Commons Attribution 4.0 International license.

10.1128/msphere.00785-21.5 FIG S2 The outcome of focusing on *nifH* in shotgun metagenomic studies. While data presented are the same as Fig. 4a to c, the confidence intervals are indicated in this plot. (a) The relationship between read counts of *nifD* and *nifH*. The gray dotted line indicates the theoretical relationship between read counts of two genes, where the number of *nifD* reads and *nifH* reads are proportional to the whole gene lengths of *nifD* and *nifH* (894 bp and 1,497 bp, respectively; the ratio is 0.597). Each sample is represented by a rectangle, which represents the 99% confidence interval of the number of *nifD* and *nifH* reads from the sample. The confidence intervals were calculated assuming that the read count follows Poisson distribution. The *x* and *y* axes are displayed in logarithmic scale [log (1 + *x*)]. Boxes deviating from the gray dotted line are indicated in red. (b) Relationship between read counts of *nifK* and *nifH*. (c) Relationship between the read counts of *nifD* and *nifK*. Download FIG S2, TIF file, 1.8 MB. Copyright © 2021 Mise et al. 2021 Mise et al. https://creativecommons.org/licenses/by/4.0/ This content is distributed under the terms of the Creative Commons Attribution 4.0 International license.

We further mapped the *nifH* reads within metagenomes onto KEGG database using rigorous (i.e., nonheuristic) Needleman-Wunsch algorithm. We classified *nifH* reads into true-*nifH*, pseudo-*nifH*, and ambiguous *nifH* reads (see Materials and Methods section for classification criteria). As suggested above, partial metagenomic sequences do not enable clear distinction between T1- and T3-*nifH* sequences (Fig. 3c). Therefore, sequences that were mapped onto both T1-NifH and T3-NifH were classified as ambiguous *nifH* reads. Here, we highlight three observations that preclude the use of *nifH* as the hallmark of diazotrophy (Fig. 4d). First, while true-*nifH* reads were dominant in some samples, others were critically affected by pseudo-*nifH* reads. In extreme cases, the abundance of *nifH* reads were exaggerated by more than 3 or 10 times owing to pseudo-*nifH* reads. This observation is consistent with a previous report on *nifH* composition in the human gut microbiome, where 444 of 524 *nifH* sequences were regarded irrelevant to nitrogen fixation (39). Second, this effect of pseudo-*nifH* was drastically different among samples, even within an environmental category. This would simply lead to inaccurate knowledge on the distribution of diazotrophs among various samples and geographic locations, which has recently been drawing attention (40, 41). Third, ambiguous *nifH* reads were dominant in many samples. This is in congruence with the results showing that partial sequences of true- and pseudo-*nifH* can be confused (Fig. 3c; Fig. S1b), meaning that simply eliminating pseudo-*nifH* reads would not be a satisfying solution. All these factors suggest that *nifH* would not be a very reliable marker in terms of specificity.

### Conclusion and outlook.

In summary, we exhaustively investigated the distribution of *nifH* genes among high-quality public genomes in RefSeq and KEGG. Using neighborhood/cooccurrence approaches, we found dozens or hundreds of “pseudo” *nifH* (i.e., *nifH* homologs unlikely to contribute to nitrogen fixation) in these databases. We also demonstrated that “pseudo” *nifH* sequences could substantially affect the metagenomic analyses of diazotrophic communities.

We envision that the prevalent use of *nifH* as the hallmark of nitrogen-fixing prokaryotes should be reconsidered. A simple and easy solution would be to focus on *nifD* or *nifK* (and their counterparts in alternative nitrogenases) instead of *nifH*, as indicated in our massive reanalysis of public metagenomes (Fig. 4a to c). It is unlikely that “pseudo-*nifD*” or “pseudo-*nifK*” sequences are prevalent, considering the proportional distributions of *nifD* and *nifK* among prokaryotic genomes (Fig. 1) and metagenomes (Fig. 4c).

Another possible solution is to assemble short-read sequences into longer contigs to enable operon-scale analysis, where pseudo-*nifH* sequences unaccompanied by *nifD* or *nifK* can be discarded. In this case, the quantitative nature of short-read sequences may be compromised: reads from true- and pseudo-*nifH* sequences might not be distinguishable (Fig. 3; Fig. S1); therefore, mapping unassembled reads onto the contigs should be hampered by nonspecific mapping (42). In this regard, simply using *nifD* or *nifK* as the marker would be a more practical choice, as it would avoid many errors that *nifH*-based analyses may incur.

## MATERIALS AND METHODS

We downloaded feature tables (i.e., annotation information of CDSs for each genome) of all genomes in the NCBI RefSeq on 21 July 2021 (19). The functional gene annotations provided in RefSeq are rigorously controlled by NCBI using PGAP, and all genomes are annotated under virtually identical (although not strictly identical) conditions. We selected genomes harboring at least one of the core genes of nitrogenase, namely, *nifD* (including *vnfD* and *anfD*), *nifH*, or *nifK* (including *vnfK* and *anfK*). We evaluated the completeness of each genome using CheckM v1.1.3 (43) with the options “lineage_wf --genes” and used only genomes with a completeness of 95% or higher. Here, CDSs labeled as pseudogenes by NCBI were discarded. To rule out the possibility that *nifD* and/or *nifK* has been overlooked by PGAP, we searched all CDSs in the *nifH*-harboring genomes for *nifD* and *nifK* using KofamScan 1.3.0 with the default parameters (22) and the database version as of April 2021. We further parsed CDS neighboring *nifH*; genes falling within 10 CDSs upstream or downstream of *nifH* were regarded as neighboring *nifH*. Only CDSs on the same strand as *nifH* were included when determining the range of the neighborhood. We classified *nifH* CDSs into the following three types: T1, *nifH* accompanied by *nifD* or *nifK* genes in their neighborhood; T2, *nifH* with *nifD* or *nifK* somewhere on the genome but not in the neighborhood; and T3, *nifH* without *nifD* or *nifK* on its genome (Fig. 2a to d). Note that some genomes have both T1 and T2, where one cluster of *nifHDK* (T1-*nifH* included) and another copy of stand-alone *nifH* (i.e., T2) coexist on one genome. We also downloaded the KEGG genomes and Kegg Orthology (KO) annotations from KEGG ftp (paywalled content; downloaded May 2021). On the basis of the KO annotations provided by KEGG, we analyzed the cooccurrences and syntenies of *nifH*, *nifD*, and *nifK* genes and classified *nifH* into three groups in the same way as we did for RefSeq.

Using FAPROTAX v1.2.4 (30), we assessed the diazotrophic activities of prokaryotic strains harboring T1-*nifH* (including those owning both T1- and T2-*nifH*), T2-*nifH* (excepting those owning both T1- and T2-*nifH*), and T3-*nifH*. Because the pipeline of FAPROTAX is designed for community-scale analysis, we generated an identity matrix as an operational taxonomic unit (OTU) table. For each type of *nifH*, we listed the taxonomic names (genus and species) of prokaryotes harboring the *nifH*, which were fed into FAPROTAX.

Next, we tested whether true- and pseudo-*nifH* sequences were distinguishable from each other. To mitigate the effect of phylogenetic bias, here we used only sequences from the members of class *Clostridia*. Furthermore, we clustered T1/2-NifH sequences at a similarity threshold of 95% using CD-HIT version 4.8.1 (44, 45). T3-NifH sequences were also similarly clustered. Hereafter in this analysis, we used only the representative sequences designated by CD-HIT (analogous to 95% operational taxonomic unit). We randomly picked subsequences of 40, 60, 80, and 100 amino acid length (10 subsequences for each length) from each of the T1/2- and T3-NifH sequences. We mapped these subsequences to the full-length T1/2-NifH and T3-NifH through an all-to-all search using the Needleman-Wunsch algorithm implemented in USEARCH v11.0.668 (with the options -search_global and -fulldp). We preformed the whole analysis with two similarity thresholds: 95% and 90%. Here, we employed global alignment, rather than local alignment (e.g., Smith-Waterman algorithm) to preclude short partial alignments. When a query from T1/2-NifH (i.e., a subsequence of T1/2-NifH) was mapped onto the T3-NifH, or vice versa, this mapping was regarded as an incorrect mapping; otherwise, the mapping was regarded as correct. We calculated the proportion of incorrect mapping for two different thresholds.

We also constructed similarity networks of partial NifH sequences. Here again, we used sequences from *Clostridia*. T1/2-NifH sequences were clustered at 95% similarity threshold to eliminate excessive redundancy in the sequences. T3-NifH sequences were clustered in the same way. The representative sequences of the clusters were subjected to MSA using the “--auto” mode of MAFFT v7.475 (46). From the constructed MSA (356 aa long), we picked 40 column-long subsequences from the MSA (for example at the position of 31 to 70 aa in the MSA, as shown in Fig. S1a in the supplemental material). The subsequences consisted of 40 aa or less, as some of them included gaps. These subsequences were subjected to all-to-all pairwise homology search using the Needleman-Wunsch algorithm implemented in USEARCH v11.0.668 (with the options -search_global and -fulldp). A sequence similarity network was constructed at a similarity threshold of 95 and 90%. If a pair of T1/2 NifH and T3-NifH were directly connected, then these two sequences were regarded “confusing.” Then we calculated the proportion of “confusing” NifH. We repeated this procedure for seven different subsequence positions in MSA: 31 to 70, 71 to 110, 111 to 150, 151 to 190, 191 to 230, 231 to 270, and 271 to 310 aa (Fig. S1). The terminus regions of MSA were occupied with many gaps and deemed unsuitable for this analysis.

Protein structures of T1-NifH and T3-NifH were predicted using AlphaFold2, a highly reliable predictor of protein structures (36). Six sequences were randomly picked from T1-NifH and from T3-NifH of genus *Clostridium* in RefSeq (listed in Fig. 4d). Each sequence was fed into the web browser interface of AlphaFold2 named ColabFold (https://colab.research.google.com/github/sokrypton/ColabFold/blob/main/AlphaFold2.ipynb; accessed on 7 August 2021) (47), which was implemented using MMseqs2 (48). The RMSD between each pair of predicted structures was calculated using Mican 2019.11.27 (49). Ward’s method was used to hierarchically cluster the structures based on RMSDs. We visualized pairwise structural alignments using Pairwise Structure Alignment toolkit (https://www.rcsb.org/alignment; accessed on 7 August 2021) hosted by the Protein Data Bank (PDB) (50).

Additionally, we assessed how pseudo-*nifH* sequences in public databases affect metagenomic analyses of environmental samples. We focused on reusable metagenomic data sets in NCBI SRA/EMBL-EBI ERA/DDBJ DRA (51) under the following environmental categories: “activated sludge metagenome,” “human gut metagenome,” “termite metagenome,” “wastewater metagenome,” and “∗ sediment metagenome” (52–64, 72). We randomly picked SRA/ERA/DRA accession numbers that satisfy the following criteria: (i) sequenced on Illumina MiSeq, HiSeq, MiniSeq, NextSeq, or NovaSeq (i.e., not subject to frameshifting read errors by Roche 454); (ii) labeled as a “WGS” (standing for whole-genome shotgun) project; and (iii) described in a peer-reviewed literature (i.e., likely to be technically sound). Two metagenomic data sets from paddy soils, one of which was labeled as “soil metagenome” on SRA/ERA/DRA (65, 66) were also used. If a project consisted of many samples, we picked 5 to 10 samples from that project.

All of the selected data sets consisted of paired-end sequences; therefore, read1 and read2 were merged using USEARCH (with the options -fastq_maxdiffs 5 -fastq_minovlen 20 -fastq_allowmergestagger). The longest consecutive subsequence with the expected number of errors below 0.5 bases was retrieved from each of the merged sequences. To increase the accuracy of sequence annotation, we retained only sequences with a length of 200 bases or more. We picked the first 2,500,000 reads from each sample and discarded samples with less than 2,500,000 filtered reads. Samples used for subsequent analyses are summarized in Table S2.

The filtered sequences were subjected to a homology search against the KEGG database to find *nifH*, *nifD*, and *nifK* reads (including their counterparts in atypical nitrogenase). First, all filtered sequences were mapped to a small database consisting only of *nifD*/*anfD* (K02586), *nifH* (K02588), *nifK*/*anfK* (K02591), *vnfD* (K22896), and *vnfK* (K22897). Here, we used DIAMOND v2.0.9.147 (67) for homology search (using blastx command with mode “sensitive”; other parameters were set default). Sequences mapped on these nitrogenase genes were again subjected to a homology search against the whole prokaryotic database of KEGG, and the numbers of queries that were annotated as *nifH* (K02588), *nifD/vnfD*/*anfD* (K02586, K22896), and *nifK/vnfK*/*anfK* (K02591, K22897) were counted. Here again we used DIAMOND, with a modification that the E-value threshold was set at 1e–10.

To accurately distinguish true-*nifH* reads and pseudo-*nifH* reads, we again mapped the translated sequences of *nifH* (K02588) reads onto the KEGG gene sequences under *nifH* (K02588) using the nonheuristic Needleman-Wunsch algorithm implemented in USEARCH. For each query, we retrieved hits with similarities above 95% of the maximum similarity. We classified K02588 reads into three groups: (i) reads mapped onto T1- and/or T2-NifH but no T3-NifH, were regarded as true-*nifH* reads; (ii) reads mapped onto only T3-NifH were regarded as pseudo-*nifH* reads; and (iii) all other reads were regarded as ambiguous reads, which could either be a true-*nifH* or a pseudo-*nifH*.

Throughout this study, taxonomic names of prokaryotes were managed using the NCBI taxonomy system (68) and TaxonKit v0.8.0 (69), and fasta and fastq files were formatted using SeqKit v0.16.1 (70). R 4.0.5 (71) was used for data visualization.

### Data availability.

Genomic and metagenomic data sets used for this study are available from NCBI RefSeq, NCBI SRA, and KEGG. Intermediate files will be made available by the authors upon request, except for the paywalled contents of KEGG, which are handled by Pathway Solutions (Tokyo, Japan).
