# Supplementary material for: Undervalued Pseudo-nifH Sequences in Public Databases Distort Metagenomic Insights into Biological Nitrogen Fixers
Source: mSphere. 2021 Nov 17;6(6):e00785-21. doi: 10.1128/msphere.00785-21 (PMC8597730; doi:10.1128/msphere.00785-21)
Supplement: TABLE S1 [file msphere.00785-21-st001.docx]

**Table S1.**

| Genome accession | Position | Gene name |
| --- | --- | --- |
| GCF_900112015.1 | 655–1527 | *nifH* |
|  | 2562–3986 | *vnfD* |
|  | 4383–5810 | *vnfK* |
| GCF_018861295.1 | 89977–90798 (negative strand) | *nifH* |
|  | 87595–89157 (negative strand) | *anfD* |
|  | 85827–87218 (negative strand) | *anfK* |
