# Supplementary figures and images for: Undervalued Pseudo-nifH Sequences in Public Databases Distort Metagenomic Insights into Biological Nitrogen Fixers
Source: mSphere. 2021 Nov 17;6(6):e00785-21. doi: 10.1128/msphere.00785-21 (PMC8597730; doi:10.1128/msphere.00785-21)

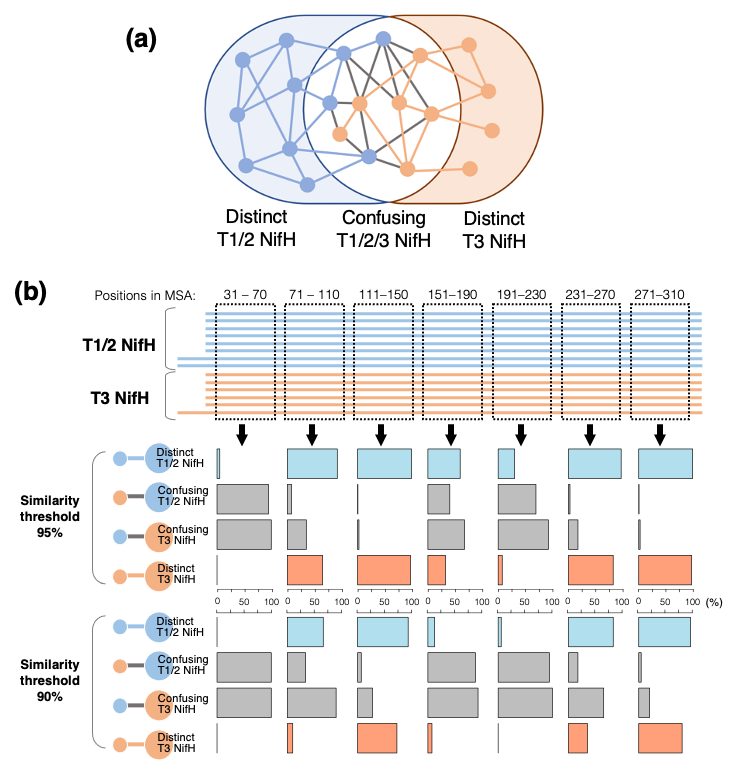

Supplement: FIG S1 [file msphere.00785-21-sf001.tif]

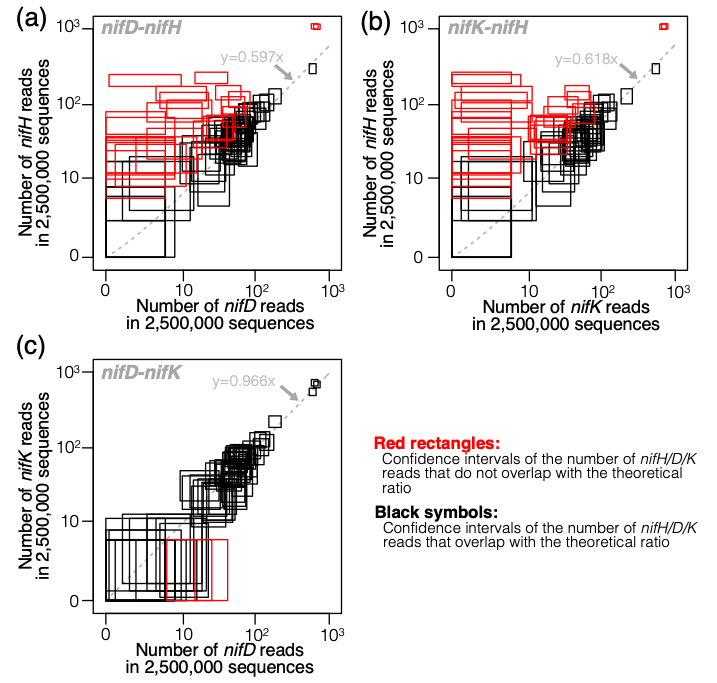

Supplement: FIG S2 [file msphere.00785-21-sf002.tif]
