# Supplementary material for: Undervalued Pseudo-nifH Sequences in Public Databases Distort Metagenomic Insights into Biological Nitrogen Fixers
Source: mSphere. 2021 Nov 17;6(6):e00785-21. doi: 10.1128/msphere.00785-21 (PMC8597730; doi:10.1128/msphere.00785-21)
Supplement: TABLE S2 [file msphere.00785-21-st002.docx]

**Table S2.**

| Environment | NCBI SRA RunID | Literature |
| --- | --- | --- |
| Wastewater | SRR13787007, SRR13787008, SRR13787011 | (1) |
| Wastewater | SRR12447079 | (2) |
| Wastewater | SRR10931996, SRR1093013 | (3) |
| Wastewater | ERR1713345, EERR1713357, ERR1713360, ERR1713381, ERR1713397 | (4) |
| Wastewater | SRR6837550, SRR6837559, SRR6837565, SRR6837580, SRR6837584 | (5) |
| Activated sludge | SRR11674002, SRR11674007, SRR11674027, SRR11674048, SRR11674052 | (6) |
| Activated sludge | SRR9261012, SRR9261017, SRR9261038, | (7) |
| Human gut | ERR4794447 | (8) |
| Human gut | SRR10680360, SRR10680603, SRR10680831 | (9) |
| Human gut | SRR5677767, SRR5677770, SRR5677781, SRR5677782, SRR5677815 | (10) |
| Human gut | SRR5813178, SRR5813186, SRR5813207 | (11) |
| Termite gut | SRR5215140, SRR5215205, SRR5215206, SRR5247064, SRR5247065, SRR5247073, SRR5247074, SRR5247076, SRR6206198, SRR6206332 | (12) |
| Paddy soil | SRR5259830, SRR5259837, SRR5259838, SRR5260069, SRR5260182, SRR5260187, SRR5260217, SRR5260272, SRR5260275 | (13) |
| Paddy soil | SRR5512136, SRR5512137, SRR5512138, SRR5512139, SRR5512141, SRR5512142, SRR5512143, SRR5512144, SRR5512146, SRR5512147 | (14) |
| Sediment | SRR12656875, SRR12656876, SRR12656880, SRR12656882, SRR12656886 | (15) |
| Sediment | SRR9649754, SRR9649755, SRR9649757, SRR9649759, SRR9649760 | (16) |

1. Peterson D, Bonham KS, Rowland S, Pattanayak CW, Klepac-Ceraj V. 2021. Comparative Analysis of 16S rRNA Gene and Metagenome Sequencing in Pediatric Gut Microbiomes. Front Microbiol 12:670336.

2. Wang M, Sun Y, Zeng Z, Wang Z. 2021. Metagenomics of wastewater phageome identifies an extensively cored antibiotic resistome in a swine feedlot water treatment environment. Ecotoxicol Environ Saf 222:112552.

3. Muller EEL, Pinel N, Laczny CC, Hoopmann MR, Narayanasamy S, Lebrun LA, Roume H, Lin J, May P, Hicks ND, Heintz-Buschart A, Wampach L, Liu CM, Price LB, Gillece JD, Guignard C, Schupp JM, Vlassis N, Baliga NS, Moritz RL, Keim PS, Wilmes P. 2014. Community-integrated omics links dominance of a microbial generalist to fine-tuned resource usage. Nat Commun 5:5603.

4. Karkman A, Berglund F, Flach C-F, Kristiansson E, Larsson DGJ. 2020. Predicting clinical resistance prevalence using sewage metagenomic data. Commun Biol 3:711.

5. Ng C, Tan B, Jiang X-T, Gu X, Chen H, Schmitz BW, Haller L, Charles FR, Zhang T, Gin K. 2019. Metagenomic and Resistome Analysis of a Full-Scale Municipal Wastewater Treatment Plant in Singapore Containing Membrane Bioreactors. Front Microbiol 10:172.

6. Singleton CM, Petriglieri F, Kristensen JM, Kirkegaard RH, Michaelsen TY, Andersen MH, Kondrotaite Z, Karst SM, Dueholm MS, Nielsen PH, Albertsen M. 2021. Connecting structure to function with the recovery of over 1000 high-quality metagenome-assembled genomes from activated sludge using long-read sequencing. Nat Commun 12:2009.

7. Pérez MV, Guerrero LD, Orellana E, Figuerola EL, Erijman L. 2019. Time Series Genome-Centric Analysis Unveils Bacterial Response to Operational Disturbance in Activated Sludge. mSystems 4: e00169-19.

8. Hildebrand F, Gossmann TI, Frioux C, Özkurt E, Myers PN, Ferretti P, Kuhn M, Bahram M, Nielsen HB, Bork P. 2021. Dispersal strategies shape persistence and evolution of human gut bacteria. Cell Host Microbe 29:1167-1176.e9.

9. Yeoh YK, Chen Z, Wong MCS, Hui M, Yu J, Ng SC, Sung JJY, Chan FKL, Chan PKS. 2020. Southern Chinese populations harbour non-nucleatum Fusobacteria possessing homologues of the colorectal cancer-associated FadA virulence factor. Gut 69:1998–2007.

10. Zuo T, Wong SH, Lam K, Lui R, Cheung K, Tang W, Ching JYL, Chan PKS, Chan MCW, Wu JCY, Chan FKL, Yu J, Sung JJY, Ng SC. 2017. Bacteriophage transfer during faecal microbiota transplantation in Clostridium difficile infection is associated with treatment outcome. Gut gutjnl-2017-313952.

11. Fukuyama J, Rumker L, Sankaran K, Jeganathan P, Dethlefsen L, Relman DA, Holmes SP. 2017. Multidomain analyses of a longitudinal human microbiome intestinal cleanout perturbation experiment. PLOS Comput Biol 13:e1005706.

12. Rossmassler K, Dietrich C, Thompson C, Mikaelyan A, Nonoh JO, Scheffrahn RH, Sillam-Dussès D, Brune A. 2015. Metagenomic analysis of the microbiota in the highly compartmented hindguts of six wood- or soil-feeding higher termites. Microbiome 3:56.

13. Hartman WH, Ye R, Horwath WR, Tringe SG. 2017. A genomic perspective on stoichiometric regulation of soil carbon cycling. ISME J 11:2652–2665.

14. Li H-Y, Wang H, Wang H-T, Xin P-Y, Xu X-H, Ma Y, Liu W-P, Teng C-Y, Jiang C-L, Lou L-P, Arnold W, Cralle L, Zhu Y-G, Chu J-F, Gilbert JA, Zhang Z-J. 2018. The chemodiversity of paddy soil dissolved organic matter correlates with microbial community at continental scales. Microbiome 6:187.

15. Petrovich M, Chu B, Wright D, Griffin J, Elfeki M, Murphy BT, Poretsky R, Wells G. 2018. Antibiotic resistance genes show enhanced mobilization through suspended growth and biofilm-based wastewater treatment processes. FEMS Microbiol Ecol 94:fiy174.

16. Zhao R, Summers ZM, Christman GD, Yoshimura KM, Biddle JF. 2020. Metagenomic views of microbial dynamics influenced by hydrocarbon seepage in sediments of the Gulf of Mexico. Sci Rep 10:5772.
