# Supplementary material for: Undervalued Pseudo-nifH Sequences in Public Databases Distort Metagenomic Insights into Biological Nitrogen Fixers
Source: mSphere. 2021 Nov 17;6(6):e00785-21. doi: 10.1128/msphere.00785-21 (PMC8597730; doi:10.1128/msphere.00785-21)
Supplement: TABLE S3 [file msphere.00785-21-st003.docx]

**Table S3**.

| Gene name  (K number) | Length (amino acid sequences) [aa] | | | | Length (gene nucleotide sequences) [bp] | | | |
| --- | --- | --- | --- | --- | --- | --- | --- | --- |
|  | Average | Q1 | Q2 | Q3 | Average | Q1 | Q2 | Q3 |
| *nifH*  (K02588) | 298 | 274 | 291 | 295 | 894 | 822 | 873 | 885 |
| *nifD*  (K02586) | 499 | 485 | 493 | 516 | 1497 | 1455 | 1479 | 1548 |
| *nifK*  (K02591) | 482 | 455 | 483 | 514 | 1446 | 1365 | 1449 | 1542 |
